# Development of lateralization of the magnetic compass in a migratory bird

**DOI:** 10.1098/rspb.2012.1654

**Published:** 2012-08-29

**Authors:** Dennis Gehring, Wolfgang Wiltschko, Onur Güntürkün, Susanne Denzau, Roswitha Wiltschko

**Affiliations:** 1 FB Biowissenschaften, J.W. Goethe-Universität Frankfurt, Siesmayerstr. 70, 60054 Frankfurt am Main, Germany; 2 Abteilung Biopsychologie, Fakultät für Psychologie, Ruhr-Universität Bochum, 44780 Bochum, Germany

**Keywords:** magnetic compass, migratory orientation, asymmetry, maturation process

## Abstract

The magnetic compass of a migratory bird, the European robin (*Erithacus rubecula*), was shown to be lateralized in favour of the right eye/left brain hemisphere. However, this seems to be a property of the avian magnetic compass that is not present from the beginning, but develops only as the birds grow older. During first migration in autumn, juvenile robins can orient by their magnetic compass with their right as well as with their left eye. In the following spring, however, the magnetic compass is already lateralized, but this lateralization is still flexible: it could be removed by covering the right eye for 6 h. During the following autumn migration, the lateralization becomes more strongly fixed, with a 6 h occlusion of the right eye no longer having an effect. This change from a bilateral to a lateralized magnetic compass appears to be a maturation process, the first such case known so far in birds. Because both eyes mediate identical information about the geomagnetic field, brain asymmetry for the magnetic compass could increase efficiency by setting the other hemisphere free for other processes.

## 1. Introduction

Cerebral lateralization refers to the division of functional processing between both hemispheres. Recent studies show that brain asymmetries are a ubiquitous vertebrate trait that possibly represents an ancient brain organization with a substantial influence on animal behaviour [1]. Individual differences in laterality can affect fitness: chimpanzees with strong hand preferences are more efficient in extracting termites [2], pigeons with prominent visual asymmetries find more grains scattered among pebbles [3], strongly lateralized parrots outperform weakly lateralized ones in cognitive problems [4] and larger language asymmetries in humans are associated with faster reading abilities [5]. Although not all studies could reveal a relationship of laterality with performance [6], it is likely that brain asymmetries modulate behavioural efficiency [7], possibly by increasing parallel processing [8] or by reducing cognitive redundancies [9]. Left–right differences of the brain require a developmental period [10], and in several systems this lateralized maturational process could be shown to correlate with improved behavioural performances [11].

In birds, the magnetic compass was found to be lateralized in favour of the right eye/left hemisphere of the brain [12]. The avian magnetic compass is an inclination compass (i.e. birds do not rely on the polarity of the magnetic field, but derive directions from the axial course of the field lines and their inclination in space) [13]. This unusual functional mode arises from the underlying physical processes: the magnetic compass of birds is based on a radical pair mechanism [14,15] that is not sensitive to the polarity of the magnetic field. Cryptochrome 1a, the most likely candidate receptor molecule for mediating directional information, is located along the discs of the outer segments of the UV-receptors [16].

An earlier study [12] showed the magnetic compass of migrating European robins, *Erithacus rubecula* (Turdidae), to be lateralized in favour of the right eye/left brain hemisphere: monocularly right-eyed birds with their left eye covered were just as well oriented as with both eyes open, whereas monocularly left-eyed birds with the right eye covered were disoriented (see also [17,18]). The same lateralization was also found in a subsequent study with migrating Australian silvereyes, *Zosterops l. lateralis* [19], and is also indicated in homing pigeons [20,21] and domestic chickens [22]. Yet, recently, Hein *et al.* [23] reported that they could not find a lateralization of the magnetic compass in two migratory species, among them the European robin. This raised the question about the reasons for the seemingly contradictory findings.

A most striking difference between the studies by Hein *et al.* [23,24] and our studies [12,17–19] was that they had tested their birds in autumn, whereas we had tested ours in spring. So the observed difference could be caused by the different testing seasons. If this was the case, it raised the question about the possible reasons. It could simply result from a maturational process of the respective neural system, or the reason for the change to asymmetry could arise from the fact that the two migrations occur during different phases in the annual cycle and are associated with different levels of hormones. A third possibility arises from the fact that the young birds in autumn, heading towards a still unknown winter quarter, are guided by innate directional information [25,26], whereas in spring birds are navigating towards a familiar goal, the breeding area [27–29]. There is evidence that the navigational ‘map’ is lateralized in favour of the left brain hemisphere [30,31], and this, in turn, could have induced a lateralization of the magnetic compass in spring.

To analyse this phenomenon and decide between these possibilities, we conducted new experiments testing young European robins in autumn during their first migration in spring and in their second autumn, where we compared their behaviour with that of a second group of robins that had been caught during their return migration in spring and hence were familiar with their winter quarter. Because there is agreement that birds are well oriented when using only their right eye, we focused on the behaviour of birds when they had to rely on their left eye alone. In the course of the study, we discovered an interesting new phenomenon that invites further analysis.

## 2. Methods

Tests took place in autumn 2010, spring 2011 and autumn 2011 in the Garden of the Zoological Institute in Frankfurt am Main (50°08′ N, 8°40′ E).

### (a) Test birds

Juvenile robins of probably Scandinavian origin were caught in September 2010 as transmigrants in the botanical garden near the institute building (autumn birds; groups A1 and A2) and kept over the winter. The photoperiod simulated the natural one during autumn (testing period 27 September–19 October 2010) until the end of December, when it was prolonged to 13 L : 11 D in the beginning of January to induce premature migratory restlessness for spring migration (testing period 12 January–14 February 2011). Other robins were caught during return migration to the breeding ground in March and April 2011 (spring birds; group Sp). These birds and group A1 were kept over the summer in a photoperiod simulating northwards migration to 62° N, a stay there and then again southwards migration. At the end of August, the photoperiod was reduced to 11.5 L : 12.5 D to promote autumn migratory activity (testing period 31 August–19 September 2011). For details on the photoperiodic treatment, see the electronic supplementary material, part 1.

### (b) Testing procedure

Testing took place in wooden houses where the local geomagnetic field was largely undisturbed (mN = 360°, 46 µT, 66° inclination). Group A2 was also tested in a field with the vertical component inverted so that the inclination was pointing upwards instead of downwards; it was produced by Helmholtz coils. We followed the protocol of the previous studies [12,18,19], with the robins tested one at a time for 1 h in funnel cages lined with thermo-paper (Blumberg Systempapiere [32]).

For monocular testing, a small aluminium cap was placed on the right eye, fixed with adhesive tape (Leukoplast) immediately before the test; it was removed immediately after the tests were finished. For the binocular control tests, birds received no treatment, because previous tests [12] had shown that any non-specific effects from covering one eye were negligible and we did not want to inconvenience the birds too much. Each bird of groups A1 and A2 was tested in these two conditions three times, alternating binocular and left-eyed tests. When this part of the study was completed in spring 2011, the birds of group A2 received their right eye caps 6 h before the beginning of the test, and were tested in the local geomagnetic field and in a field with the vertical component inverted (table 1). For the experiments in autumn 2011, the birds of group A1 and group Sp were tested three times binocularly and monocularly left-eyed, then monocularly left-eyed again after the right eye had been occluded for 6 h. A last binocular test was to show that the birds were still in their migratory phase at the end of the study.

**Table 1.** Orientation of European robins in autumn and spring in the various test conditions. Condition: Bi, binocular control; L, monocularly left-eyed; 6peL, monocularly left-eyed after 6 h pre-exposure with the right eye covered; 6peLvi, as for 6peL, but tested in a magnetic field with the vertical component inverted; Bi final, control test at the end of the series. *N*, number of birds; *n*, number of tests per bird; med. *r*_b_, median of the vector lengths per bird, reflecting the intra-individual variance; *α*_N_ and *r*_N_, direction (in parentheses if not significant) and length, respectively, of the grand mean vector, with asterisks at r_N_ indicating a significant directional preference (Rayleigh test [33]); *Δ*Bi, angular difference to the binocular control (in parentheses if the compared sample is not significantly oriented) with asterisks indicating significance of the difference in directions (indicated by ^d^) and in variance. *p < 0.05; **p < 0.01; ***p < 0.001; n.s., not significant. For vectors of the individual birds, see electronic supplementary material, tables in part 2.

group	season	condition	*N*	*n*	med. *r*_b_	*α*_N_	*r*_N_	*Δ*Bi
*robins caught in autumn 2010*								
group A1	autumn 2010	Bi	12	3	0.59	193°	0.68**	
		L	12	3	0.80	185°	0.84***	−8° n.s.
	spring 2011	Bi	12	3	0.93	357°	0.73***	
		L	12	3	0.53	(273°)	0.25 n.s.	(−83°)**
	autumn 2011	Bi	11	3	0.90	183°	0.80***	
		L	11	3	0.58	(317°)	0.14 n.s	(+134°)**
		6peL	11	3	0.48	(201°)	0.13 n.s.	(+18°)*
		Bi final	11	1	—	188°	0.88***	+5° n.s.
group A2	spring 2011	Bi	12	3	0.92	347°	0.74***	
		L	12	3	0.60	(53°)	0.33 n.s.^.^	(+66°) n.s.
		6peL	12	3	0.81	21°	0.92***	+34° n.s.
		6peLvi	12	2	0.97	177°	0.89***	−170° *** ^d^
*robins caught in spring 2011*								
group Sp	autumn 2011	Bi	11	3	0.74	189°	0.73**	
		L	11	3	0.53	(177°)	0.19 n.s	(−12°)*
		6peL	11	3	0.67	(353°)	0.24 n.s	(+164°)*
		Bi final	11	1	—	179°	0.81***	−10° n.s.

### (c) Data analysis and statistics

The data analysis followed our standard procedure [12,17–19]: the thermo-paper was removed from the cage, and from the distribution of the activity, the heading of that test was determined blind. The three (or two) headings of each bird in each condition were comprised in the respective mean vector (*α*_b,_ *r*_b_) of that bird. On the basis of the birds' mean headings, the grand mean vectors (*α*_N_, *r*_N_) were calculated and tested by the Rayleigh test for significant directional preferences [33]. The data of the test conditions were compared with the respective binocular controls using the Watson–Wheeler test [33] for differences in direction (if *r*_N_ > 0.65) and the Mann–Whitney test for differences in variance. For a detailed description of the data analysis and statistical treatment, see the electronic supplementary material, part 1.

## 3. Results

Table 1 summarizes the results in the various test conditions numerically; the vectors of the individual birds are listed in the electronic supplementary material, part 2.

### (a) Behaviour in first autumn and spring

**Figure 1.**
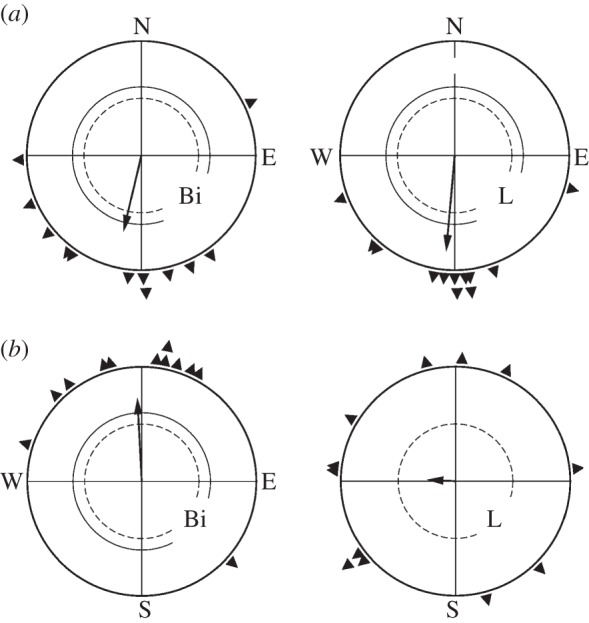
Orientation of the same twelve young robins (group A1) in (*a*) their first autumn and (*b*) the following spring in the geomagnetic field. Bi, tested binocularly (control); L, tested monocularly left-eyed. The triangles at the periphery of the circle indicate the mean headings of individual birds, the arrows represent the grand mean vectors in relation to the radius of the circle = 1, with the two inner circles representing the 5% (dashed line) and 1% (solid line) significance border of the Rayleigh test [33].

**Figure 2.**
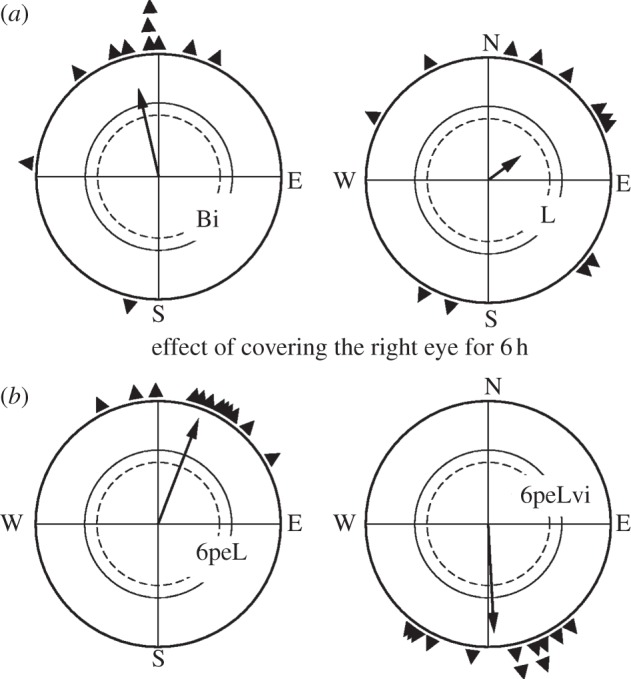
Orientation of robins of group A2 in spring. The tests took place in the local geomagnetic field unless otherwise indicated. Bi, binocularly tested (control); L, tested monocularly left-eyed; 6peL, tested monocularly left-eyed after 6 h pre-exposure with the right eye covered; 6peLvi, same as for 6peL, but tested in a magnetic field with the vertical component inverted. Symbols as in figure 1.

In the first part of the study, we tested the birds of group A1 caught in autumn in both seasons in the geomagnetic field. With both eyes open, they were significantly oriented in their seasonal appropriate migratory direction in both seasons. When relying on their left eye alone, they were also significantly oriented in their migratory direction in autumn, but no longer in spring (figure 1). The other autumn birds, group A2, showed the same disoriented behaviour in spring when they had to rely on their left eye only (figure 2*a*). That is, in autumn, the birds in our study showed no lateralization, whereas in spring, we again found the same strong lateralization in favour of the right eye/left brain hemisphere as in our previous studies [12,17–19].

With the group A2, we analysed the lateralization of the magnetic compass in spring in more detail, in particular whether it would be affected if the input from the right eye was disrupted. Hence, we pre-exposed the birds to the monocularly left-eyed situation by covering their right eye for 6 h prior to testing. Now the left-eyed birds proved significantly oriented in their northerly migratory direction in the geomagnetic field, and their behaviour was indistinguishable from that recorded under binocular control conditions (*p* > 0.05). When they were tested in a magnetic field with the vertical component reversed and the inclination was upwards instead of downwards, they reversed their headings, a behaviour demonstrating that this orientation of the left-eyed birds was controlled by the inclination compass as migratory orientation normally is [13] (figure 2*b*). This means that the lateralization of the magnetic compass was flexible: it could be removed by forcing the birds to rely on their left eye alone—blocking the input of the dominant eye abolished the asymmetry of magnetic compass orientation within just a few hours.

### (b) Behaviour in second autumn

In the test in second autumn, we compared the behaviour of group A1 that had been caught in autumn the year before (and was thus unfamiliar with the wintering area) with that of group Sp caught in spring on their return journey from the winter quarters.

**Figure 3.**
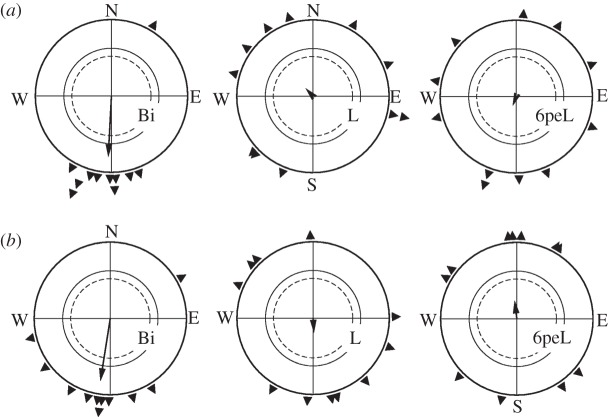
Orientation of robins during their second autumn migration. All tests took place in the local geomagnetic field. (*a*) Robins of group A1 caught in autumn the year before; they were unfamiliar with their winter quarter (same birds as in figure 1); (*b*) robins of group Sp caught in spring during return migration; they were familiar with their goal. Bi, binocularly tested (control); L, tested monocularly left-eyed; 6peL, tested monocularly left-eyed after 6 h pre-exposure with the right eye covered.

The results of the respective tests are given in figure 3. There was no difference between the two groups (*p* > 0.05): the birds of group A1 as well as birds of group Sp were disoriented when they had to rely on their left eye alone, regardless of whether they were familiar with the goal or not. At the same time, the lateralization seemed to have become stronger, as covering the right eye for 6 h now failed to remove it.

## 4. Discussion

Our data from the second autumn indicate that the origin of the direction of migration—flying innate courses versus navigation to a familiar goal—does not influence the lateralization of the magnetic compass. Its lateralization in favour of the right eye/left brain hemisphere appears to be the result of a maturation process. Apparently, as the young birds grow older, the neural architecture of the respective brain centres specializes in a way that magnetic directional information is based only on input from the right eye processed in the left hemisphere of the brain. We cannot exclude, though, that differences in hormone state may be additionally involved. Gonadal hormonal levels have been shown to modify lateralization [34], but because we did not test the birds in their second spring, this question must remain open. Hence, while lateralization is maturation-dependent, its fixation could still be regulated by hormones.

Lateralization, in particular of the visual system, is widespread among birds [1]. However, a lateralized function that develops only slowly with time has not been known in this class of vertebrates, although similar phenomena have been observed in lateralized systems in the human brain (e.g. in connection with handedness or face recognition) [10,11]. Our findings additionally show that the maturation of the asymmetry of the magnetic compass proceeds through an intermediate phase during first spring in which it can easily be reversed by covering the right eye for just a few hours. In the following autumn, asymmetry becomes more stabilized, although we cannot exclude that a longer monocular occlusion could reinstall an ability to use the magnetic compass left-eyed. The flexibility observed in spring suggests that the lateralization does not take place at the receptor level, but higher up in the brain where magnetic compass information is processed. This is in agreement with the observation that cryptochrome 1a, the most likely receptor molecule, is present in both eyes alike [16].

The fact that initially a mere 6 h of obstructing right eye input can modulate the asymmetry suggests that stimulation-induced modifications of synaptic strengths play a crucial role. Our findings point to the existence of competitive and modifiable synaptic interactions between inputs from both eyes, possibly along the ascending visual system. In birds, visual projections that reach the forebrain constitute the tecto- and the thalamofugal pathways. Synaptic convergence from both eyes takes place in the nucleus rotundus of the tectofugal pathway [35,36] and also in the visual Wulst of the thalamofugal system [37], with the latter being discussed as relevant for magnetic compass orientation [38]. Thus, monocular obstruction of right eye input for several hours could increase the synaptic weight of left eye input at tecto- and thalamofugal convergence zones of both eyes. As a result, the left eye could then successfully feed magnetic compass information into the processing system. During the subsequent maturational period, however, plasticity of neuronal wiring seems to be tuned down and can no longer be altered by a few hours of biased visual input. The processes leading to synaptic stabilization of the lateralized avian magnetic compass are unknown and invite further analysis.

An additional open question is the possible advantage of the asymmetry of the magnetic compass system. In contrast to vision and hearing, where the differential input between right and left eyes or ears conveys additional information, magnetic field input from both eyes is redundant, because both eyes provide the identical information on the direction of the magnetic field. Thus, the right eye superiority of the magnetic compass could free the capacity of circuits reached by the other eye, and could thus increase neural efficiency during tasks that demand the simultaneous but different use of both hemispheres [8].
